# Antitumor Effect and Gut Microbiota Modulation by Quercetin, Luteolin, and Xanthohumol in a Rat Model for Colorectal Cancer Prevention

**DOI:** 10.3390/nu16081161

**Published:** 2024-04-13

**Authors:** Álvaro Pérez-Valero, Patricia Magadán-Corpas, Suhui Ye, Juan Serna-Diestro, Sandra Sordon, Ewa Huszcza, Jarosław Popłoński, Claudio J. Villar, Felipe Lombó

**Affiliations:** 1 Research Group BIONUC (Biotechnology of Nutraceuticals and Bioactive Compounds), Departamento de Biología Funcional, Área de Microbiología, Universidad de Oviedo, 33006 Oviedo, Spainsernadjuan@uniovi.es (J.S.-D.); cjvg@uniovi.es (C.J.V.); 2 Instituto Universitario de Oncología del Principado de Asturias (IUOPA), 33006 Oviedo, Spain; 3 Instituto de Investigación Sanitaria del Principado de Asturias (ISPA), 33006 Oviedo, Spain; 4 Department of Food Chemistry and Biocatalysis, Wrocław University of Environmental and Life Sciences, Norwida 25, 50-375 Wrocław, Poland; sandra.sordon@upwr.edu.pl (S.S.); ewa.huszcza@upwr.edu.pl (E.H.); jaroslaw.poplonski@upwr.edu.pl (J.P.)

**Keywords:** flavonoid, intraperitoneal, colorectal cancer, rat model, gut microbiota

## Abstract

Colorectal cancer stands as the third most prevalent form of cancer worldwide, with a notable increase in incidence in Western countries, mainly attributable to unhealthy dietary habits and other factors, such as smoking or reduced physical activity. Greater consumption of vegetables and fruits has been associated with a lower incidence of colorectal cancer, which is attributed to their high content of fiber and bioactive compounds, such as flavonoids. In this study, we have tested the flavonoids quercetin, luteolin, and xanthohumol as potential antitumor agents in an animal model of colorectal cancer induced by azoxymethane and dodecyl sodium sulphate. Forty rats were divided into four cohorts: Cohort 1 (control cohort), Cohort 2 (quercetin cohort), Cohort 3 (luteolin cohort), and Cohort 4 (xanthohumol cohort). These flavonoids were administered intraperitoneally to evaluate their antitumor potential as pharmaceutical agents. At the end of the experiment, after euthanasia, different physical parameters and the intestinal microbiota populations were analyzed. Luteolin was effective in significantly reducing the number of tumors compared to the control cohort. Furthermore, the main significant differences at the microbiota level were observed between the control cohort and the cohort treated with luteolin, which experienced a significant reduction in the abundance of genera associated with disease or inflammatory conditions, such as *Clostridia UCG-014* or *Turicibacter*. On the other hand, genera associated with a healthy state, such as *Muribaculum*, showed a significant increase in the luteolin cohort. These results underline the anti-colorectal cancer potential of luteolin, manifested through a modulation of the intestinal microbiota and a reduction in the number of tumors.

## 1. Introduction

Cancer, a global health challenge, encompasses a group of disorders arising from abnormal and uncontrolled cell proliferation with invasive characteristics [1,2]. Colorectal cancer (CRC) stands as the third most prevalent malignancy worldwide, with over 1.93 million newly reported cases and 935,173 deaths in 2020, securing its place as the third leading cause of mortality in both, males and females, globally [3,4,5]. Notably, CRC has emerged as a predominant cancer in Western countries, contributing to 10% of worldwide cancer incidence and 9.4% of cancer-related deaths [3].

This higher prevalence of CRC in Western countries is attributed to the aging population and the prevalence of unhealthy dietary practices, including high intakes of saturated fats and nitrosamines, along with a low consumption of fruits and vegetables. Additional contributing factors include smoking, low physical activity, and obesity, which together affect colonic mucosal health [6,7]. Another factor affecting the prevalence of this disease is the time of its onset. CRC typically appears late in life, as its progression requires several genetic mutations, resulting in a higher incidence in the adult population over 55–60 years old [8].

The initiation of CRC involves genetic (such as loss of *APC*, *TP53*, or *KRAS* genes or in the mismatch repair genes *MLH1*, *MSH2*, *MSH6*, or *PMS2*, due to direct mutations, or the *BRAF* gene due to microsatellite instability-associated point mutations), and epigenetic alterations (such as silencing of *MLH1* gene by promoter hypermethylation, CpG islands hypermethylation silencing at promoter regions in several genes, or miRNA methylation) in the stem cells located at the base of the colon crypts, which globally generate different subtypes of CRC cases (based on chromosomal instability, microsatellite instability, or CpG-island methylator phenotype) as those following the adenoma/carcinoma pathway or the serrated pathway [9,10]. These modifications affect tumor suppressor genes and oncogenes, leading to the transformation of normal stem cells into neoplastic stem cells [11]. The resulting genetic and epigenetic changes contribute to the loss of genomic and/or epigenomic stability, leading to tumor lesions in the colon, including aberrant crypt foci (ACF), adenomas, and serrated polyps. This process is a pivotal event in the initiation and development of CRC, both at the pathophysiological and molecular levels [12,13].

In CRC, most tumors originate from a polyp formed from an ACF. This polyp progresses to an early adenoma of less than 1 cm, which then evolves to an advanced adenoma exceeding 1 cm in size. Ultimately, the advanced adenoma becomes transformed into a malignant tumor (adenocarcinoma), which can acquire metastatic capabilities [14].

The neoplastic progression caused by ACF within the colonic mucosa can be modulated to attenuate or prevent its progression via the presence of a variety of nutraceutical compounds in the colon lumen, particularly polyphenols and short-chain fatty acids from prebiotic fiber fermentation [8]. Polyphenols are the largest group of plant bioactive chemicals known in nature. They have been associated with numerous health benefits, such as cancer prevention [15]. Within polyphenols, flavonoids are a large family of nutraceuticals widely distributed in plants, including edible plants [15,16,17,18,19]. Although flavonoids are primarily known for their antioxidant attributes, findings from in vitro and in vivo studies underline their ability as anti-inflammatory and immunomodulatory bioactives [20], as well as robust anticancer compounds [21,22,23,24]. The molecular mechanisms underlying the anticancer effects of flavonoids are not yet fully elucidated [24]. Nevertheless, their recognized functions include the modulation of reactive oxygen species (ROS)-scavenging enzymatic activities. Furthermore, flavonoids are involved in the regulation of critical cellular processes, such as cell cycle arrest, apoptosis induction, autophagy, as well as suppression of cancer cell proliferation and invasiveness [20,21,22,23,24,25,26].

Within the extensive family of flavonoids, quercetin, luteolin, and xanthohumol possess anti-CRC activity [1,27,28]. Quercetin, which has been shown to be safe for addition to foods [29], shows an inhibition of cell viability in CT26 and MC38 colon cancer cells. It induces apoptosis through the mitogen-activated protein kinases (MAPKs) pathway and modulates the expression of epithelial-mesenchymal transition (EMT) markers, including E-cadherin, N-cadherin, β-catenin, and SNAI1 [27].

Luteolin, a safe chemopreventive agent [30] characterized by its potent antioxidant and anti-inflammatory effects, is remarkably effective in CRC and its associated complications. It attenuates the expression of nitric oxide synthase (iNOS) and cyclooxygenase-2 (COX-2). Additionally, luteolin suppresses the expression of matrix metalloproteinase-2 (MMP-2) and matrix metalloproteinase-9 (MMP-9) to address CRC-related issues [1]. The antitumor activity of luteolin shows synergy in combination with current antitumor drugs, such as 5-fluorouracil (5-FU), against human CRC cell lines in vitro, which makes this flavonoid highly interesting for future in vivo co-therapy studies [31].

On the other hand, xanthohumol, whose administration in mice has been proved as safe [32], is able to reduce proliferation in HT-29 CRC cells [28,33]. Moreover, xanthohumol decreases the expression of several drug efflux genes. This property makes it an interesting candidate for combination therapy with other anticancer chemotherapeutic agents, presenting a potential strategy to mitigate drug resistance by inhibiting drug efflux transporters [28,34]. Luteolin and xanthohumol have also demonstrated good in vitro antitumor activity against several human colon cancer cell lines [35].

Most studies available in the literature have tested flavonoids against CRC cell lines or in murine CRC models through oral administration as nutraceuticals. However, oral administration affects the bioavailability of flavonoids mainly due to their limited solubility and changes carried out by gut microbiota in their chemical skeletons, such as hydrolysis at ring C. In contrasts, intraperitoneal administration allows the final molecule to be actually absorbed to the portal circulation directly from the peritoneal cavity to the plasma via a network of capillaries in the peritoneum that arrive at the liver, where they undergo phase II metabolism (sulfation and methylation) and finally reach the digestive tract via bile component [36,37,38]. In contrast, the use of flavonoids as therapeutic drugs, intraperitoneally (or via intravenous administration), instead of nutraceuticals to combat CRC, has rarely been studied [39].

The present investigation was designed to examine the possible antitumor effects of quercetin, luteolin, and xanthohumol administered intraperitoneally in an animal model of CRC, specifically *Rattus norvegicus* F344. In this study, CRC was chemically induced by a combination regime including azoxymethane (AOM) and dextran sodium sulphate (DSS). The animals were systematically analyzed for several parameters, including body weight, caecum weight, hyperplastic Peyer’s patches count, colon length, and tumor number. Additionally, the composition of the gut microbiota was examined within the four cohorts (control, quercetin, luteolin, and xanthohumol), revealing notable distinctions among them.

## 2. Materials and Methods

### 2.1. Animals and Experimental Design

A total of 40 male Fischer 344 rats were maintained in the Animal Facilities at the University of Oviedo (authorized facility No. ES330440003591). All rat experiments were approved by the ethics committee of the Principality of Asturias (authorization code PROAE 14/2022).

The rats (five weeks old) were randomly divided into four cohorts of ten individuals each and fed ad libitum (2014 Teklad Global 14% Protein Rodent Maintenance Harlan diet feed, Harlan Laboratories, Barcelona, Spain). This feed contained 14.3% protein, 4% fat, 48% carbohydrates, 22.1% fiber, and 4.7% ashes. The rats were placed in a room with constant temperature (21 °C) and humidity on a 12:12 h dark/light cycle throughout the experiment.

Cohort 1 was injected intraperitoneally with 200 µL of phosphate buffered saline (PBS) (VWR International, Radnor, PA, USA) as control. Cohort 2 was injected with 25 mg per kg body weight (mg/kg/bw) of quercetin (Apollo Scientific, Bredbury, UK). Cohort 3 was injected with 25 mg/kg/bw of luteolin (Fluorochem, Hadfield, UK). Cohort 4 was injected with 25 mg/kg/bw of xanthohumol. All the flavonoids were dissolved in PBS and injected into the animals 3 days a week during the 18 weeks of the experiment.

Xanthohumol was purified following a modified procedure described previously [40]. The same batch of spent hops, stored in high-density polyethylene (HDPE) industrial barrels and closed under a nitrogen atmosphere, was used. The purification modification involved only the initial extract preparation step, as it was fully completed at the Department of Food Chemistry and Biocatalysis, Wrocław University of Environmental and Life Sciences laboratories. Eighteen kilograms of spent hops were extracted with ninety L of acetone in 0.2 kg:1.4 L batches, each made in a 2 L Erlenmeyer flask shaken for 3 h on a rotary shaker (120 rpm). The formed pulp was vacuum-filtered on Whatman filter paper no. 4 and concentrated using a laboratory rotary evaporator. The combined extracts were further subjected to polyphenol precipitation and Sephadex LH 20 column chromatography steps, resulting in 20.233 g of Xanthohumol (>98% purity with HPLC).

### 2.2. Colorectal Cancer Induction and Monitoring

One week after the animals arrived at the animal facility, the injections started. After one week receiving the flavonoids, CRC was induced in eight rats from each cohort. The two other rats were kept free of CRC induction as absolute control animals, used as sentinels for detecting any potential side effect of the treatments. CRC induction was carried out in those eight rats of each cohort using AOM (Sigma-Aldrich, Madrid, Spain) dissolved in sterile saline (0.9% NaCl) at a concentration of 2 mg/mL. This AOM solution was injected intraperitoneally at a final concentration of 10 mg/kg/bw. The AOM treatment was repeated seven days after the first injection (weeks 2 and 3). The two absolute control animals received sterile saline solution in both injections.

In weeks 4 and 15, the eight rats of each cohort (those treated with AOM) received drinking water ad libitum for 7 days containing 3% and 2% DSS (40.000 g/Mol, VWR), respectively. This ulcerative colitis step was repeated twice because it enhances the pro-carcinogenic effect caused by AOM administration.

The rats were weighed once a week during the 18 experimental weeks. At the end of the experiment, the rats were sacrificed using pneumothorax.

### 2.3. Tissue Samples

The rats were anesthetized (isoflurane) and submitted to euthanasia (pneumothorax) at week 18. The small intestine was removed fresh and the hyperplastic Peyer’s patches were counted.

The caecums were weighed immediately after sacrifice using a precision scale and then frozen at −20 °C.

Finally, the colon was opened longitudinally and washed with PBS before keeping it in 4% formaldehyde at 4 °C. Fixed colons were meticulously examined in order to count the number of tumors.

### 2.4. Genomic DNA Extraction and 16S Ribosomal RNA Sequencing for Gut Microbiota Analysis

A metagenomics analysis of caecal stool specimens was conducted employing the Pathogen Detection Protocol facilitated by the E. Z. N. A.^®^ Stool DNA Kit (Ref. D4015-02, VWR, Madrid, Spain). The caecal specimens, after being thawed in ice for a duration of 30 min, underwent the extraction of 200 mg of feces from the midsection of the caecum, which was then placed in a 25 mL tube for subsequent protocol adherence. Subsequently, 200 μL of genomic DNA was isolated and quantified using a BioPhotometer^®^ (Eppendorf, Madrid, Spain).

The processed DNA samples were frozen at −20 °C for subsequent analysis via the amplification and sequencing of the variable regions V3 and V4 of the 16S ribosomal RNA gene using Illumina MiSeq (Microomics Systems, Barcelona, Spain). The Illumina Miseq sequencing 300 × 2 methodology was employed, with amplification conducted after 25 PCR cycles. Quality control measures included the incorporation of a negative control for DNA extraction and a positive mock community control. This approach facilitated the characterization and quantification of microbial alpha and beta diversity, along with the examination of taxonomic profiles spanning from phylum to species levels.

### 2.5. Bioinformatics Analysis

The phylotype data served as the basis for calculating alpha diversity metrics, facilitating the analysis of the microbial community diversity. The assessment encompassed community richness, denoted by the count of observed operating taxonomic units (OTUs), representing distinct phylotypes within a community. Additionally, community evenness was evaluated using Pielou’s evenness index, quantifying the numerical equality of phylotypes within a community, considering both their abundance and number. The determination of alpha diversity metrics also included indices such as Chao1 (indicative of species richness), Simpson (reflecting biodiversity levels), and Shannon (representing species diversity).

Beta diversity metrics, comparing microbial community structure among cohorts, were computed based on both phylotype and phylogenetic data. A principal coordinate analysis (PCoA) employing unweighted Unifrac distance, a phylogenetic qualitative measure, was executed to discern differences in beta diversity among the microbial communities.

### 2.6. Statistical Methods

In the context of the metagenomics analysis, comparisons of alpha diversity were executed utilizing a linear model with an appropriate distribution, specifically the negative binomial model for Chao1, beta regression for Simpson, and a linear model for Shannon diversity. Beta diversity distance matrices were employed to compute PCoA and construct ordination plots using the R software version 4.2.0. The assessment of community structure significance among groups was conducted through the permutational multivariate analysis of variance (Permanova) test.

The differential relative abundance of taxa was scrutinized using a linear model based on the negative binomial distribution or ANOVA. The statistical analyses involved the utilization of BiodiversityR version 2.14-1, PMCMRplus version 1.9.4, RVAideMemoire version 0.9-8, and vegan version 2.5-6 packages.

For additional comparisons, group data were expressed as mean ± standard error of the mean (SEM). The Shapiro–Wilk test was used for calculating the Gaussian distribution of the different variables. One-way ANOVA (Holm–Šídák multiple comparison test) was used for comparisons between flavonoid-treated cohorts and the control cohort following a Gaussian distribution. In the case of no Gaussian distribution, one-way ANOVA (Kruskal–Wallis test) was used for determining the statistical differences among cohorts. The graphical representation of the data was executed using GraphPad Prism software (version 9.0.2, GraphPad Software, San Diego, CA, USA).

In the case of the number of tumors, the ROUT method (Q = 5%) was used to identify outliers. Rat number 8 in the Cohort 3 was identified as an outlier (31 tumors) and removed for the statistical analysis.

In each case, a *p*-value < 0.05 was considered statistically significant (* *p* < 0.05; ** *p* < 0.005; *** *p* < 0.0005; **** *p* < 0.0001).

## 3. Results

### 3.1. Effect of Quercetin, Luteolin, and Xanthohumol Administration on Body Weight

**Figure 1:**
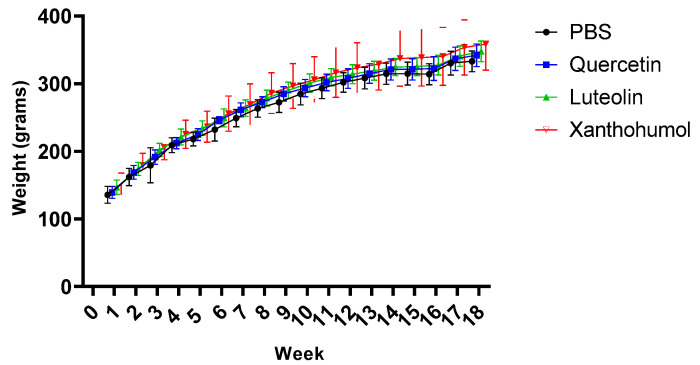
Evolution of body weight throughout the entire experiment (18 weeks, X axis) of the eight rats with CRC induction in the four cohorts. Body weight was measured every week. When the animals were sacrificed, the mean value for Cohort 1 was 332.9 ± 15.3 g, for Cohort 2 it was 341.9 ± 16.6 g, for Cohort 3 it was 347.8 ± 15.2 g, and for Cohort 4 it was 358.5 ± 38.8 g.

Animals in all four cohorts showed constant weight gain over the 18 experimental weeks. CRC was induced in rats 1–8 of each cohort. The two AOM challenges for CRC induction took place at weeks 2 and 3, and both DSS events were carried out at weeks 4 and 15. Figure 1 shows a slightly slowdown in weight gain after the second DSS event, with a notably recovery in weight gain from week 16 until the end of the experiment. However, no obvious slowdown in weight gain was observed after the first DSS event during week 4. Furthermore, rats numbered 3 and 7 in Cohort 2 (quercetin) died after the first DSS event, in week 5, as a consequence of the episode of transitional ulcerative colitis, a pro-inflammatory step necessary to increase the final number of tumors generated by AOM.

On the other hand, in the absolute control animals (rats 9 and 10 in each cohort), the increase in body weight was constant throughout the experiment since these animals did not receive AOM or DSS. In summary, none of the treatments caused a strong increase or reduction in body weight.

### 3.2. Effect of Quercetin, Luteolin, and Xanthohumol Administration on Hyperplastic Peyer’s Patches

The number of Peyer’s patches in the small intestine was quantified when the animals were sacrificed. Peyer’s patches contain large numbers of lymphocytes and can become hyperplastic. These lymphoid nodules are easily observable as elongated thickenings in the intestinal mucosa, measuring a few millimeters in length.

**Figure 2:**
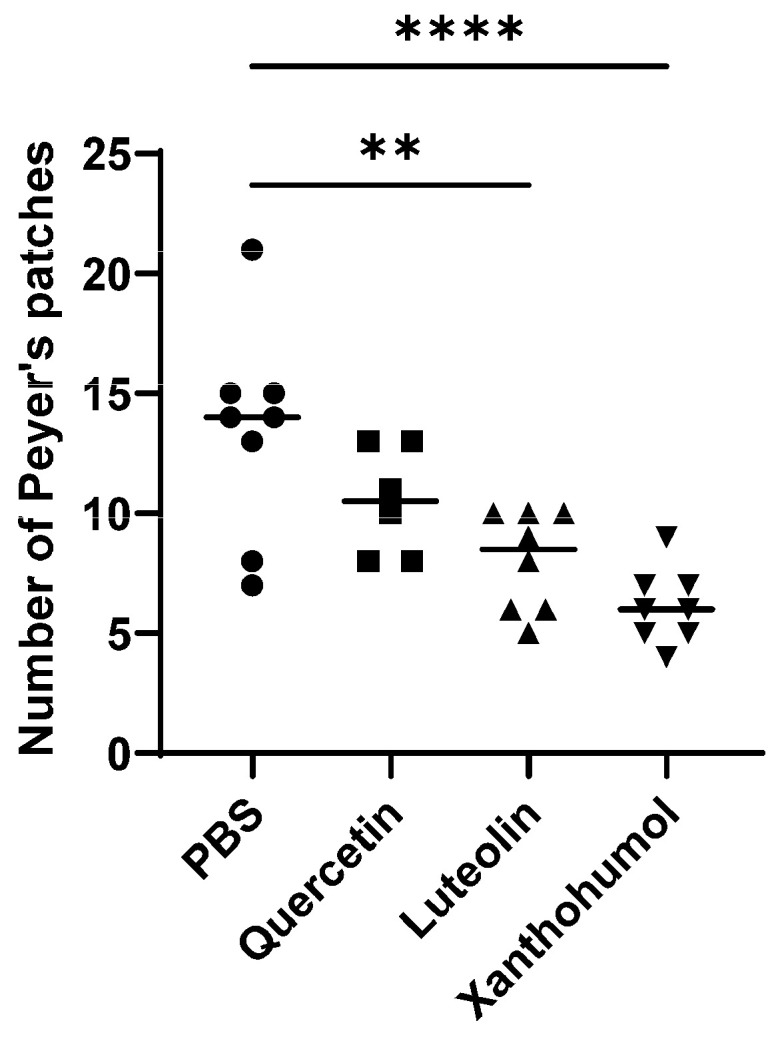
Mean number of hyperplastic Peyer’s patches in the small intestine of animals with induced CRC from each cohort (rats 1 to 8 in the 4 groups). The median value of each cohort is represented as a horizontal line within the corresponding box plots. Asterisks indicate statistically significant differences (** *p* < 0.005; **** *p* < 0.0001) (ANOVA test).

In this study, the differences in the mean values of Peyer’s patches were statistically significant between Cohort 3 (luteolin) and Cohort 1 (PBS), and between Cohort 4 (xanthohumol) and Cohort 1, where a drastic decrease of 40.2% and 54.2% in the number of Peyer’s patches, respectively, was observed (Figure 2), a sign of anti-inflammatory protective bioactivities due to luteolin and xanthohumol administration.

### 3.3. Effect of Quercetin, Luteolin, and Xanthohumol Administration on Caecum Weight

No statistically significant differences were observed in the mean values of caecum weight when comparing the animals induced for CRC within the control cohort, with those subjected to treatments with quercetin, luteolin, or xanthohumol (Figure S1). Likewise, comparable non-significant distinctions were observed between the absolute control animals of the control cohort and the flavonoid-treated cohorts.

### 3.4. Effect of Quercetin, Luteolin, and Xanthohumol Administration on Colon Length

No statistically significant differences were found in mean colonic length measurements between the control cohort and the cohorts treated with quercetin, luteolin, or xanthohumol (Figure S2). Similarly, no significant differences were observed between the absolute control animals of the control cohort and those of the flavonoid-treated cohorts.

### 3.5. Effect of Quercetin, Luteolin, and Xanthohumol Administration on the Number of Tumors

**Figure 3:**
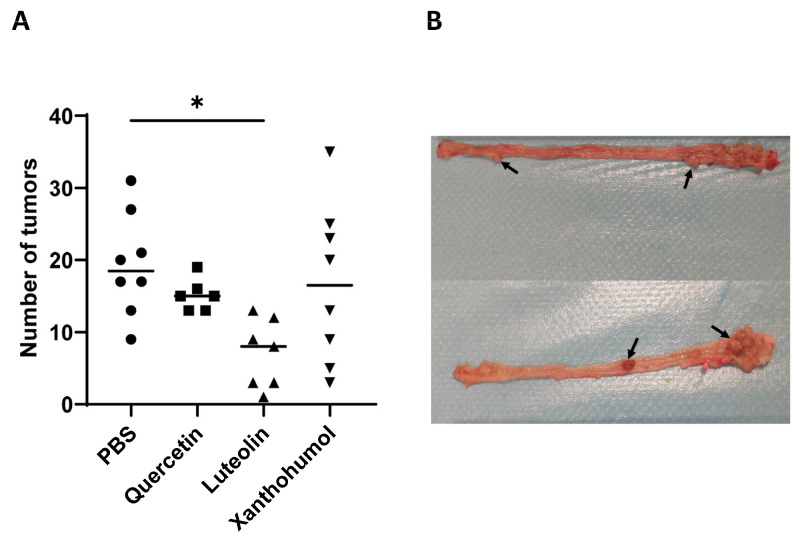
(**A**) Average number of colon tumors in each cohort (rats 1 to 8 in the 4 cohorts). The average number of tumors showed a statistically significant decrease in the case of Cohort 3 (luteolin) compared to the control Cohort 1. The median value of each cohort is represented as a horizontal line within the corresponding box plots. (**B**) Image showing two representative colons of rats in which CRC was induced. Some tumors are highlighted with black arrows. Asterisks indicate statistically significant differences (* *p* < 0.05) (ANOVA test).

The colon mucosa in each of the rats was analyzed to determine the number of tumors. A statistically significant difference was only observed between rats in Cohort 3 (luteolin) and Cohort 1 (control). Cohort 3 showed a drastic 63.9% reduction in the number of tumors (Figure 3), indicating a potent antitumor potential for luteolin. The number of tumors was also reduced in Cohort 2 (quercetin) and Cohort 4 (xanthohumol), but these reductions were not statistically significant (Figure 3). No tumors were found in the colonic mucosa of the absolute control animals (rats 9 and 10) of each cohort, as expected, as those animals were not submitted to AOM tumor induction, and the flavonoid treatments were not carcinogenic.

### 3.6. Effect of Quercetin, Luteolin, and Xanthohumol Administration on the Gut Microbiota

#### 3.6.1. Alpha and Beta Diversity

Alpha diversity metrics, which encompass diversity within each animal sample, were assessed by measuring richness (observed operational taxonomic units or OTUs) and evenness within microbial communities. Additionally, alpha diversity was comprehensively assessed using statistical indexes such as Chao1, Simpson, and Shannon. Figure S3 illustrates box plot representations of these diversity measures. When comparing CRC-induced animals from the PBS cohort with the corresponding animals from each of the flavonoid treatment cohorts, no statistically significant differences were discerned for any of the alpha diversity measures. This absence of significant differences indicates a lack of alterations in microbial alpha diversity in CRC-induced animals under the influence of the flavonoid treatments.

**Figure 4:**
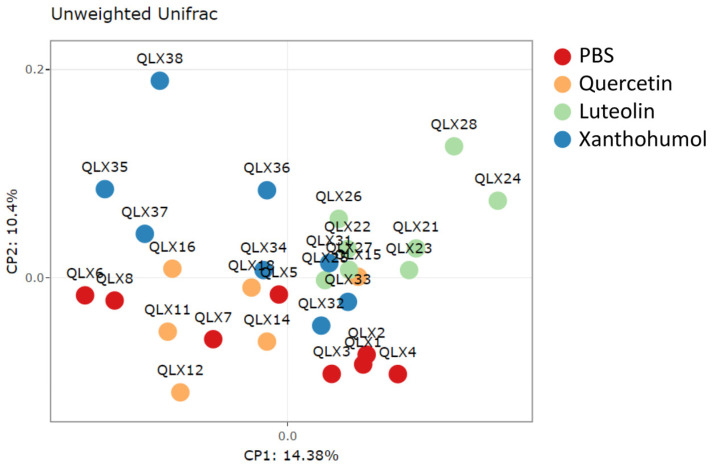
PCoA plot showing structural variations within microbial communities measured using the unweighted Unifrac beta diversity index. Each dot labeled with “QLX” and a numerical identifier indicates an individual participant in the experiment. Specifically, red dots represent CRC-induced animals in the PBS-treated cohort, orange dots belong to CRC-induced quercetin-treated animals, green dots correspond to CRC-induced luteolin-treated animals, and blue dots represent CRC-induced individuals of the xanthohumol-treated cohort.

The unweighted Unifrac beta diversity index, representing diversity among samples, was calculated to assess differences between groups with respect to species complexity. The PCoA plot, represented in Figure 4, visually shows the structural variations within the microbial communities of CRC-induced rats. Differences in beta diversity were evident in the comparison between the PBS control group and the quercetin-, luteolin-, or xanthohumol-treated cohorts, as illustrated in Table 1. Moreover, discernible differences in beta diversity were observed between the quercetin and both the luteolin and xanthohumol cohorts, as well as between the two cohorts undergoing treatments with luteolin or xanthohumol (Table 1), indicating that these two treatments caused more similar alterations in colon microbiota populations, far from PBS and quercetin conditions.

#### 3.6.2. Taxonomic Profile

Metagenomic analysis of the cecal microbiota in CRC-induced animals revealed significant differences between various taxonomic levels and between different experimental cohorts. In general, *Bacillota* (formerly *Firmicutes*) and *Bacteroidota* (formerly *Bacteroidetes*) emerge as the predominant phyla, being consistently observed in all cohorts and comparisons. The relative abundance of the different phyla in the CRC-induced animals of the four cohorts shows variations that depend on the specific treatment and the comparisons made (Table 2).

**Figure 5:**
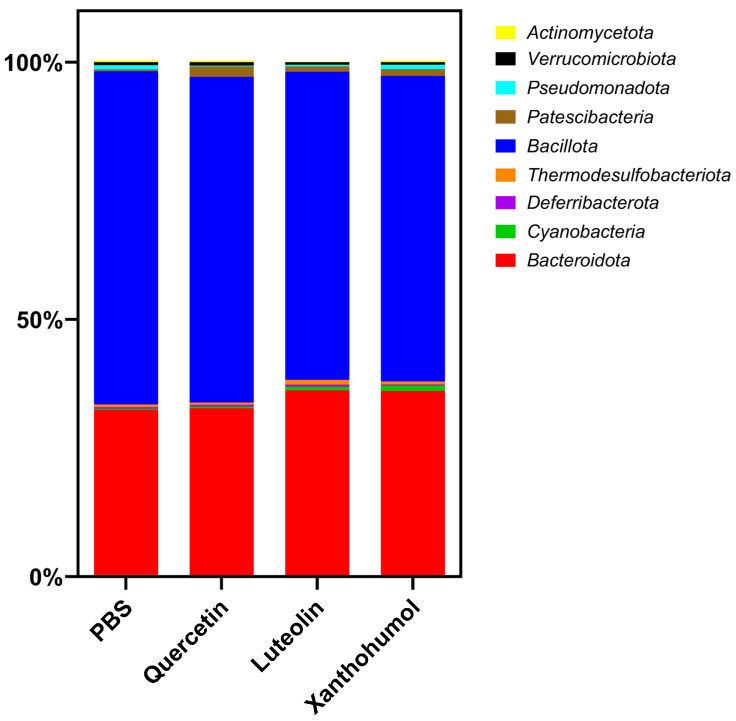
Average gut microbiota composition at the phylum level for the CRC-induced rats belonging to the four cohorts.

The quercetin cohort showed a statistically significant increase compared to the PBS cohort in the abundance of the phylum *Patescibacteria* (0.35% vs. 2.06% in the PBS cohort, *p*-value 0.00581) and a significant decrease in the phylum *Pseudomonadota* (0.80% vs. 0.15% in the PBS cohort, *p*-value 0.00913). In the case of the luteolin cohort, compared to the PBS cohort, notable variations were observed between different phyla. Specifically, the phyla *Actinomycetota* and *Bacillota* experienced reductions after luteolin treatment (0.33% vs. 0.10% in the luteolin cohort and 64.78% vs. 59.87% in the luteolin cohort, respectively) with associated *p*-values of 0.0001 and 0.02865, respectively. In contrast, the abundance of the phylum *Bacteroidota* exceeded that of the PBS cohort (31.01% vs. 36.08% in the luteolin cohort (*p*-value 0.00179). Finally, the xanthohumol cohort demonstrated an increase in the average percentage of the phyla *Bacteroidota* (32.01% in the PBS cohort vs. 35.80%), *Cyanobacteria* (0.27% in the PBS cohort vs. 1.06%), and *Patescibacteria* (0.35% in the PBS cohort vs. 1.28%), with associated *p*-values of 0.00415, 0.00167, and 0.04696, respectively. On the contrary, the percentage of the phylum *Bacillota* decreased from 64.78% in the PBS cohort to 59.41% in the xanthohumol cohort (*p*-value 0.01227). These variations are graphically represented in Figure 5.

**Figure 6:**
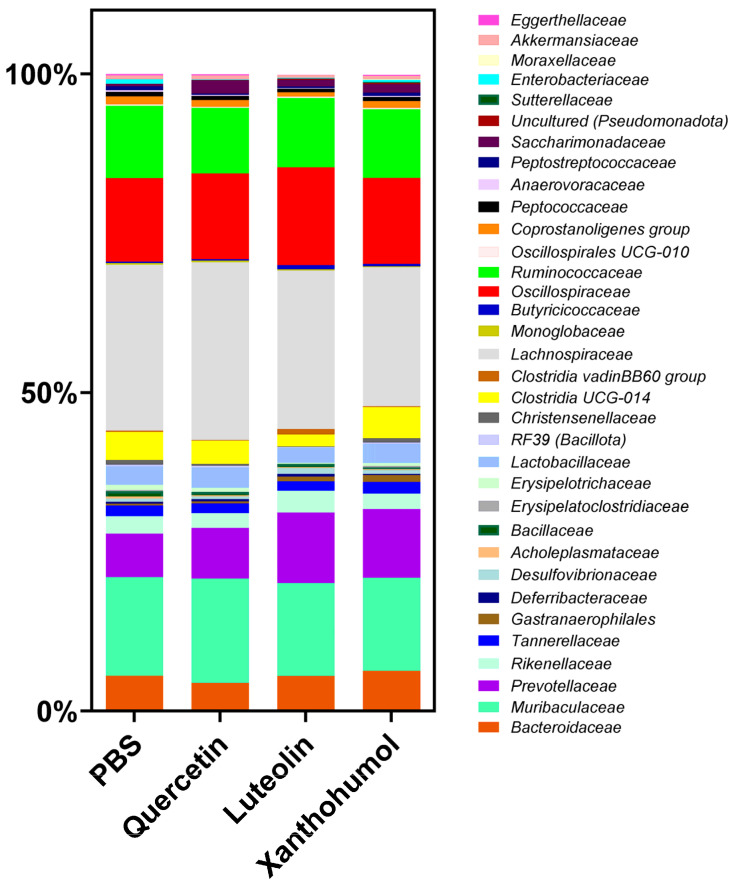
Differences in the average composition of the gut microbiota at the family level for the CRC-induced rats belonging to the four cohorts.

At the taxonomic family level, the variations in abundance between families of CRC-induced animals in the four cohorts depend on the specific treatment administered and the comparisons made. The predominant families in all cohorts include *Lachnospiraceae*, *Muribaculaceae*, *Oscillospiraceae*, and *Ruminococcaceae*, as shown in Figure 6. Substantial differences were seen between the cohorts subjected to flavonoid treatments and the control cohort. Families exhibiting statistically significant variations are described in Table 3.

Significant differences were identified between PBS and quercetin cohorts in the families *Saccharimonadaceae*, which presented an increase from 0.35% to 2.06%, and *Enterobacteriaceae*, which decreased from 0.69% to 0.07%. The most pronounced disparities were observed between the PBS cohort and the luteolin cohort, where a significant decrease was observed in the families *Eggerthellaceae* (0.24% vs. 0.05%), *Erysipelotrichaceae* (0.82% vs. 0.13%), *Christensenellaceae* (0.77 vs. 0.09%), and *Anaerovoracaceae* (0.26% vs. 0.10%). On the contrary, an increase was observed in the families *Clostridia* vadinBB60 group (0.19% vs. 0.88%) and *Butyricicoccaceae* (0.23% vs. 0.61%).

Significant differences were also found between the PBS cohort and the xanthohumol cohort, revealing an increase in the abundance of the families *Gastranaerophilales*, *Saccharimonadaceae*, and *Sutterellaceae* (0.27% vs. 1.06%, 0.35% vs. 1.28%, and 0.01% vs. 0.16%, respectively). Finally, a significant increase in the abundance of the *Prevotellaceae* familly was observed between the PBS cohort and the luteolin and xanthohumol cohorts (6.83% vs. 11.09% and 6.83% vs. 10.80%, respectively).

**Figure 7:**
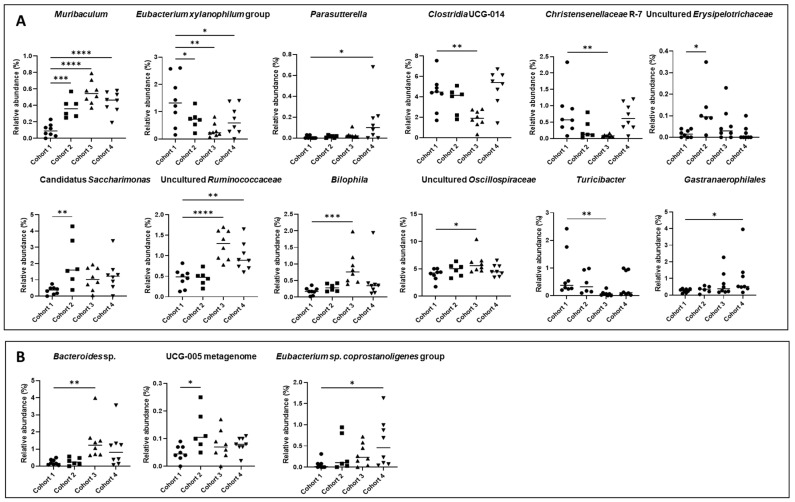
(**A**) Significant taxonomic variations at the genus level observed between rats with CRC-induced within the PBS cohort (Cohort 1) and the cohorts subjected to treatment with quercetin, luteolin, or xanthohumol (Cohorts 2, 3, and 4, respectively). (**B**) Significant taxonomic differences at the species level observed between rats with CRC induced within the PBS cohort (Cohort 1) and the cohorts subjected to treatment with quercetin, luteolin, or xanthohumol (Cohorts 2, 3, and 4, respectively). The median value of each cohort is represented as a horizontal line within each cohort. Asterisks indicate statistically significant differences (* *p* < 0.05; ** *p* < 0.005; *** *p* < 0.0005; **** *p* < 0.0001) (ANOVA test).

At the taxonomic genus level, statistically significant differences were also observed through the comparative analysis of the PBS cohort and the cohorts subjected to flavonoid treatments, as shown in Figure 7A. The main differences were found between PBS (Cohort 1) and luteolin (Cohort 3). Genera such as *Clostridia UCG-014*, *Turicibacter*, and *Christensenellaceae R-7* showed significant reductions exclusively in luteolin (Cohort 3) (from 4.37%, 0.77%, and 0.76% in the PBS cohort vs. 1.88%, 0.07%, and 0.08% in the luteolin cohort, respectively). Furthermore, the *Eubacterium xylanophilum* group experienced a significant decrease in all flavonoid-treated cohorts (from 1.40% in the PBS cohort to 0.71%, 0.31%, and 0.69% in quercetin, luteolin, and xanthohumol, respectively). In contrast, the relative abundance of *Muribaculum* increased significantly in all cohorts (0.24% in the PBS cohort vs. 0.37% in the quercetin cohort, 0.56% in the luteolin cohort, and 0.44% in the xanthohumol cohort). Similarly, an uncultured genus of the family *Ruminococcaceae* increased significantly in luteolin and xanthohumol cohorts (from 0.45% in the PBS cohort to 1.27% and 0.98% in the luteolin and xanthohumol cohorts, respectively), while the genera *Bilophila* and another uncultured genus of the family *Oscillospiraceae* showed significant increases only in the luteolin cohort (from 0.18% and 3.99% in the PBS cohort to 0.88% and 6.10% in the luteolin cohort, respectively). Finally, the genera *Parasutterella* and *Gastranaerophilales* experienced a significant increase in the xanthohumol cohort (from 0.01% and 0.20% in the PBS cohort to 0.16% and 0.71 in the xanthohumol cohort, respectively), and an uncultured genus of the family *Erysipelotrichaceae* and a Candidatus *Saccharimonas* experienced an increase only in the quercetin cohort (from 0.02% and 0.35% in the PBS cohort to 0.13% and 2.06% in the quercetin cohort, respectively). All of these notable variations are described in Table 4.

At the species level, notable variations in relative abundance were also observed. However, the majority of these species remain unidentified (Figure 7B). Three species were identified that exhibit notable differences between the PBS control cohort and the flavonoid-treated cohorts, none of which belong to the aforementioned genera. It is worth highlighting the abundance of *Bacteroides* sp., which exhibited an increase in the luteolin cohort compared to the control cohort (0.23% vs. 1.45%). On the contrary, *Eubacterium* sp. *coprostanoligenes* group showed a higher abundance in the xanthohumol cohort in relation to the control cohort (0.06% vs. 0.58%). Finally, a species identified as *UCG-005 metagenome* showed an increase in its abundance in the quercetin cohort compared to the PBS cohort (0.05% vs. 0.13%).

## 4. Discussion

This work has evaluated the antitumor potential of the flavonoids quercetin, luteolin, and xanthohumol in an animal model where CRC was induced using AOM and DSS. These three flavonoids have already been shown to have antitumor effects in the treatment of CRC through oral administration as nutraceuticals [1,27,28,33,34,41]. Here, we performed intraperitoneal administration of the compounds to study their effects as pharmaceutical compounds against CRC. A total of forty rats participated in this study, divided into four cohorts: Cohort 1, which received PBS instead of flavonoids (used as a control), Cohort 2 (treated with quercetin), Cohort 3 (treated with luteolin), and Cohort 4 (treated with xanthohumol). Within each cohort, eight rats were induced for CRC, while two rats did not receive AOM nor DSS (absolute controls, which were used as sentinels for detecting potential side effects of the flavonoid treatments). The CRC-induced animals from the flavonoid-treated cohorts were compared with the corresponding animals from the control cohort at different levels (physical and metagenomics parameters).

Animals from the four cohorts showed a constant weight gain throughout the 18 experimental weeks and no significant differences were found between the flavonoid-treated cohorts and the control cohort. However, after the first DSS event (week 4), rats numbered 3 and 7 in Cohort 2 (quercetin) died as a result of intense colitis. Furthermore, a slowdown in weight gain was observed after the second DSS event (week 15) in all cohorts, with rapid recovery from week 16 until the end of the experiment (Figure 1). Once the experiment was completed at week 18, the 38 surviving animals were sacrificed.

In order to evaluate the anti-inflammatory potential of these compounds, quantification of hyperplastic Peyer’s patches was performed in all rats. These protuberances within the mucosal structures of the small intestine have an abundance of lymphocytes and undergo hyperplasia in response to alterations in the digestive tract. These hyperplastic changes imply a pro-inflammatory immune status in the animals [42]. Luteolin and xanthohumol demonstrated the potential to reduce the number of hyperplastic Peyer’s patches after intraperitoneal administration, with this second compound being the most effective (Figure 2). This result can be easily explained since luteolin and xanthohumol are well-known anti-inflammatory compounds [43,44]. In the case of quercetin, although a reduction in the average number of hyperplastic Peyer’s patches was observed, no statistically significant variations were found when compared with the control cohort (Figure 2). Like many other flavonoids, quercetin has also been shown to be an anti-inflammatory compound [45]; however, an in vitro study showed that a significant amount of quercetin can remain adhered to the wall of the small intestine of the rat, reducing its availability [46], which could explain, in our case, its lower anti-inflammatory effect.

Regarding caecum weight (Figure S1), as expected, no significant variations were observed between cohorts since flavonoids, although they are prebiotics, are not fermentable compounds like inulin or other polysaccharide-type prebiotics [47,48,49] and therefore do not contribute to the gain of microbial mass in this organ. However, flavonoids and their derived metabolites do have the ability to modulate the composition of the gut microbiota. This modulation occurs by inhibiting certain undesirable bacterial strains or increasing concentrations of beneficial genera [50].

At the colon level, no significant differences were found in terms of their lengths (Figure S2). In contrast, the number of colon tumors showed a significant decrease in the luteolin cohort (average of 7 tumors) when compared to the PBS control cohort (average of 19 tumors) (Figure 3), demonstrating the antitumor potential of luteolin when administered intraperitoneally at the tested dose (25 mg/kg/bw). These results are in accordance with another study carried out in mice to which luteolin was administered intraperitoneally in AOM-induced animals, where the average number of tumors was reduced from 9.4 in the control cohort to 4.2 in the group that received luteolin [51]. In vitro, luteolin has shown high antitumor activity against the human CRC cell lines HCT116, HT-29, and T84, even with a synergistic effect when administered together with the commercial antitumor 5-FU [35]

Regarding quercetin, it has been shown to exert an anti-CRC effect when administered orally at a concentration of 2% in the diet [52]. However, lower doses of quercetin were ineffective in reducing tumor numbers [53]. In this study, quercetin was administered intraperitoneally at 25 mg/kg/bw, but no significant reduction in tumor count was observed (Figure 3). This inconsistency may be attributed to the different bioavailability profile associated with intraperitoneal administration compared to oral administration, which would make the dose ineffective in achieving the desired antitumor activity. Finally, in previous works, xanthohumol has demonstrated antitumor potential in CRC cell lines [35,54,55], displaying a better antitumor effect than that of current pharmacological drugs in use for chemotherapy of this cancer, such as 5-FU [35]. In contrast to the antitumor properties exhibited by xanthohumol in CRC cell lines, the performance of this compound in this rat CRC model closely reflects (at the level of tumor numbers) that of the control group that did not receive any flavonoid treatment (Figure 3). These observations suggest that xanthohumol may not be a potent antitumor agent in this context, despite its notable anti-inflammatory activity evidenced by the reduction in the number of hyperplastic Peyer’s patches.

Regarding the transport of intraperitoneally administered flavonoids, the sub-mesothelial stratum of the peritoneum supports an intricate but effective vascular network comprising blood and lymphatic vessels [56]. Compounds within the visceral peritoneum traverse the venous system, gaining entry into the portal vein. The peritoneum is richly perfused with blood capillaries, thus providing an optimal surface for the bidirectional exchange of pharmaceutical agents between the peritoneal cavity and plasma. Molecules introduced via portal circulation undergo integration with systemic circulation after hepatic transit, leading to rapid first-pass metabolism of the administered substances [37]. The liver functions as a pivotal organ in phase II metabolism, facilitating the conjugation of flavonoids through processes such as sulfation and methylation. Subsequently, the resulting metabolites are excreted as bile components back to the small intestine and reach the intestinal microbiota, undergoing deconjugation and subsequent reabsorption [38].

A fundamental disparity arises in flavonoid processing between oral and intraperitoneal intake. Orally administered flavonoids undergo alterations catalyzed by intestinal phase II enzymes, producing conjugated metabolites. These metabolites enter the portal circulation and subsequently reach the liver where additional modifications take place [38]. In contrast, flavonoids administered intraperitoneally directly access the portal system on their route to the liver [37]. This difference makes intraperitoneal administration a faster route for flavonoid absorption. Furthermore, the complexity of the flavonoid absorption process, whether by oral or intraperitoneal administration, could explain the differences observed in the effect of flavonoids against CRC between CRC cell lines and CRC animal models due to the absence of this entire circulatory network in the in vitro tests on cell lines, where flavonoids are absorbed directly by cells with a smaller amount (or absence) of enzymatic modifications in their structure.

The processing of flavonoids by the intestinal microbiota, after deconjugation, leads to the production of various hydroxyphenylacetic acids [57,58,59]. For example, quercetin undergoes metabolic transformations that lead to the formation of 2-(3,4-dihydroxyphenyl) acetic acid, 2-(3-hydroxyphenyl) acetic acid, and 3,4-dihydroxybenzoic acid from its B ring. Simultaneously, the A ring produces phloroglucinol, 3-(3,4-dihydroxyphenyl) propionic acid, and 3-(3-hydroxyphenyl) propionic acid [60]. Within the intestinal environment, these compounds can also undergo initial alterations through the fission of the C ring, involving various metabolic pathways, followed by subsequent dihydroxylation reactions [61]. In the case of luteolin, the compounds generated due to microbiota metabolism are 3-(4′-hydroxyphenyl) propionic acid and 3-(3′,4′-dihydroxyphenyl) propionic acid, which are formed by cleavage of the C ring. In both cases, the release of phloroglucinol occurs as a byproduct of this process [59,62]. For xanthohumol, the metabolite resulting from the action of the gut microbiota is 8-prenylnaringenin, a well-known potent phytoestrogen [62].

All of these compounds have the ability to shape the intestinal microbiota in different ways, which will affect their antitumor activity. To study the taxonomic variations between the different cohorts of this work, the composition of the intestinal microbiota was determined by metagenomic 16S rRNA sequencing of cecal content. In terms of alpha diversity, richness and evenness remained similar in the different cohorts (Figure S3), while interesting differences were found with respect to beta diversity between the groups (Figure 4). The beta diversity measure suggests that the PBS cohort and the quercetin cohort have a similar community structure although significant differences were found between them (Table 1). In the case of the luteolin cohort, large differences were found when compared with the rest of the cohorts (*p*-value 0.002 for all comparisons) (Table 1), indicating a less related community structure with the rest of the cohorts. Finally, the xanthohumol cohort also showed significant differences with all the other cohorts, especially with luteolin, which is in accordance with the good antitumor performance observed in the case of luteolin but not with xanthohumol. These findings suggest that quercetin, luteolin, and xanthohumol exhibit the ability to modulate the community structure of the gut microbiota in the CRC-induced animals, with special emphasis on the notable differences observed in luteolin treatment. These distinctions are evident when clustering and plotting samples on a heatmap using genus abundance data (Figure S4).

At the phylum level, *Bacillota* was significantly reduced in both luteolin and xanthohumol cohorts, while *Bacteroidota* was significantly increased in both of them. It has been previously shown that the abundance of the phylum *Bacillota* decreased in the lumen of a CRC rat model compared to healthy rats [63], supporting our observations in the case of the luteolin treatment, which has been shown to possess the best antitumor effect among the flavonoids analyzed in this work. However, this dysbiosis was also observed in the xanthohumol cohort, which showed a tumor count similar to that of the control cohort. In contrast, the phylum *Actinomycetota* was reduced only in the luteolin cohort (Table 2), and this lower abundance has been associated with healthy rats [63].

At the family level, *Prevotellaceae* was the most abundant family in all cohorts, being extremely lower in the CRC-induced rats of PBS cohort (control) compared to the luteolin and xanthohumol cohorts, especially in the case of the luteolin cohort, where the abundance was almost the double (Table 3). Other authors have observed a similar result, where the *Prevotellaceae* family has been significantly increased in healthy rats compared to CRC rats [63]. In this study, based on the results obtained, the luteolin cohort is the healthiest group, which supports that the abundance of the *Prevotellaceae* family may be related to a better state of health. Paradoxically, this family was also overrepresented in the xanthohumol cohort, where the number of tumors was similar to the one observed in the PBS cohort. However, xanthohumol demonstrated a great anti-inflammatory effect, which may explain the high abundance of this family. The *Erysipelotrichaceae* family was also overrepresented in the control cohort and significantly reduced in the luteolin cohort (Table 3). The high abundance of this family has been linked to CRC status [63]. Regarding the family *Christensenellaceae*, a notable reduction was observed in the luteolin cohort (Table 3). A decrease in the abundance of this family has been postulated to be advantageous to health, based on a study among African American patients with colorectal cancer (CRC) [64], which is consistent with our findings. However, a different study reported elevated levels of *Christenellaceae* in healthy controls compared to individuals with CRC [65]. These incongruent trends can be explained since it has been observed that the association of *Christensenellaceae* with CRC depends on the type of specific mutation present [66,67]. On the other hand, as shown in Table 3, we observed a significant decrease in the abundance of the family *Enterobacteriaceae* in the luteolin cohort, which may be related to an unhealthy state (due to the presence of lipopolysaccharide in this family, as well as other virulence factors), since this family is associated with CRC due to the production of the organic compound trimethylamine n-oxide [68]. Other families, such as *Eggerthellaceae* and *Anaerovoracaceae,* showed a reduction in the luteolin cohort, while the *Clostridia vadinBB60* group and *Butyricicoccaceae* experienced an increase. This dysbiosis observed at the family level can be correlated with an improvement in health status (due to a higher production of anti-inflammatory short-chain fatty acids, among other factors such as lower production or presence of virulence factors) after the administration of luteolin as a therapeutic intervention [69].

At the genus level, *Muribaculum* increased significantly in all the flavonoid-treated cohorts, especially in the luteolin cohort (Table 4 and Figure 7). A significant abundance in this genus has been positively associated in CRC mouse models, as bacteria of this genus have demonstrated the ability to maintain intestinal homeostasis by utilizing mucin monosaccharides [70]. The genus *Bilophila* was overrepresented in the luteolin cohort (Table 4 and Figure 7). It is well known that *Bilophila wadsworthia* converts taurine to the toxic metabolite hydrogen sulfide, an activity associated with CRC [71], and this pathogenic gut population has been shown to be inhibited after administration of functional meats enriched in flavonoids [8]. However, this species did not experience dysbiosis and the differences observed in *Bilophila* may be associated with other species in this genus. In contrast, a notable reduction in the abundance of the genus *Christensenellaceae R-7* was observed exclusively within the luteolin cohort (Table 4 and Figure 7). This reduction is notable, particularly considering that the prevalence of this genus has previously been correlated with healthy conditions [72]. In the case of *Clostridia UCG-014,* which has been commonly reported as pro-inflammatory bacteria [73], a significant reduction in the luteolin cohort was observed, compared to the control cohort (Table 4 and Figure 7). This is supported by a study conducted in a colitis-associated CRC mouse, where a decrease in this genus was observed within the CRC cohort that received natural shikonin, which was found to be a preventive agent for this neoplasia [74]. The genus *Eubacterium xylanophilum* group was reduced in all flavonoid-treated cohorts, especially in the luteolin cohort (Table 4 and Figure 7). Controversially, this genus was found to be increased in a CRC-mice cohort fed with rice bran, which improved the CRC condition [75]. Regarding the *Oscillospiraceae* and *Ruminococcaceae* families, a notable increase in the abundance of an uncultured genus within each family was found, which was particularly evident in the luteolin cohort (Table 4 and Figure 7). The taxon “*Candidatus* Saccharimonas” was significantly increased in the quercetin cohort, while the beneficial genus *Parasutterella* [76] remained more abundant in the xanthohumol cohort. Regarding the genus *Turicibacter*, it has been associated with various diseases, such as acute appendicitis [77,78]. Moreover, *Turicibacter* was increased in other studies with CRC-induced mice [78,79], which supports the observed reduction in its abundance here in the luteolin cohort.

## 5. Conclusions

In summary, this study has elucidated the antitumor potential of the flavonoid luteolin in a CRC rat model when administered intraperitoneally. Both luteolin and xanthohumol have shown the ability to significantly reduce the number of hyperplastic Peyer’s patches in the small intestine, which is an inflammation biomarker. The luteolin cohort experienced a significant reduction in the number of colon tumors compared to the control cohort. In addition, a metagenomic study has been carried out to analyze the possible differences in the microbiota of the different cohorts, finding the main differences (with respect to the control cohort) in the luteolin cohort, where some bacterial families and genera associated with a good prognosis (such as those ones generating antitumor short-chain fatty acids like propionate or butyrate, which inhibit histone deacetylases, inducing apoptosis only in tumor colonocytes) experienced an increase, while other groups of harmful bacterial decreased (such as those ones involved in inflammation onset, stimulation of colonocytes proliferation via β-catenin activation, generation of reactive oxygen or nitrogen species (RONS) able to cause DNA mutations on colonocytes, or in the activation of procarcinogens) [69,80,81]. These results show the ability of flavonoids, particularly luteolin, to modulate the intestinal microbiota in an animal model for CRC to contribute to an improvement in the health of individuals. In addition, it confirms the effectiveness of the intraperitoneal administration of flavonoids, as drugs.

## Figures and Tables

**Table 1:** Significance (*p*-value) between cohorts in terms of beta diversity using the Permanova test. Cohort 1: PBS administration; Cohort 2: quercetin administration; Cohort 3: luteolin administration; Cohort 4: xanthohumol administration.

	Cohort 1	Cohort 2	Cohort 3
**Cohort 2**	0.037		
**Cohort 3**	0.002	0.002	
**Cohort 4**	0.0084	0.0075	0.002

**Table 2:** Average percentage composition of the gut microbiota at the phylum level in CRC-induced animals for the four cohorts studied. Underlined phyla indicate significant differences (ANOVA test) between at least one of the cohorts treated with flavonoids (Cohorts 2, 3, and 4) and the control cohort (Cohort 1). Percentages marked by asterisks indicate that the cohort shows a statistically significant difference compared to the control within a specific phylum, along with the corresponding level of statistical significance. Asterisks indicate statistically significant differences (* *p* < 0.05; ** *p* < 0.005; **** *p* < 0.0001).

	Cohort 1 (%)	Cohort 2 (%)	Cohort 3 (%)	Cohort 4 (%)
*Actinomycetota*	0.33	0.30	0.10 ****	0.26
*Bacteroidota*	32.01	32.40	36.08 **	35.80 **
*Cyanobacteria*	0.27	0.34	0.69	1.06 *
*Deferribacterota*	0.31	0.34	0.43	0.19
*Thermodesulfobacteriota*	0.53	0.43	0.95	0.60
*Bacillota*	64.78	63.33	59.87 *	59.41 *
*Patescibacteria*	0.35	2.06 **	1.07	1.28 *
*Pseudomonadota*	0.80	0.15 **	0.34	0.87
*Verrucomicrobiota*	0.62	0.67	0.48	0.52

**Table 3:** Average percentage composition of statistically significant intestinal microbiota families in CRC-induced animals for the four cohorts studied. Underlined families indicate significant differences (ANOVA test) between at least one of the cohorts treated with flavonoids (Cohorts 2, 3, and 4) and the control cohort (Cohort 1). Percentages marked with asterisks indicate that the cohort shows a statistically significant difference compared to the control cohort within a specific family, along with the corresponding level of statistical significance. Cohort 1: PBS administration; Cohort 2: quercetin administration; Cohort 3: luteolin administration; Cohort 4: xanthohumol administration. Asterisks indicate statistically significant differences (* *p* < 0.05; ** *p* < 0.005; *** *p* < 0.0005; **** *p* < 0.0001).

	Cohort 1 (%)	Cohort 2 (%)	Cohort 3 (%)	Cohort 4 (%)
*Eggerthellaceae*	0.24	0.16	0.05 ****	0.18
*Bacteroidaceae*	5.26	4.21	5.42	6.12
*Muribaculaceae*	15.50	16.42	14.61	14.61
*Prevotellaceae*	6.83	7.93	11.09 ****	10.80 ****
*Rikenellaceae*	2.76	2.34	3.40	2.38
*Tannerellaceae*	1.63	1.49	1.49	1.88
*Gastranaerophilales*	0.27	0.34	0.69	1.06 **
*Deferribacteraceae*	0.31	0.34	0.43	0.19
*Desulfovibrionaceae*	0.53	0.43	0.95	0.60
*Acholeplasmataceae*	0.30	0.17	0.15	0.14
*Bacillaceae*	0.85	0.53	0.49	0.30
*Erysipelatoclostridiaceae*	0.17	0.05	0.04	0.24
*Erysipelotrichaceae*	0.82	0.60	0.13 ***	0.43
*Lactobacillaceae*	2.93	3.20	2.35	2.97
*RF39* (*Bacillota*)	0.25	0.29	0.11	0.24
*Christensenellaceae*	0.77	0.28	0.09 ****	0.67
*Clostridia UCG-014*	4.37	3.64	1.88	4.94
*Clostridia vadin *BB60 group	0.19	0.09	0.88 *	0.11
*Lachnospiraceae*	26.07	27.94	24.93	21.84
*Monoglobaceae*	0.25	0.23	0.20	0.13
*Butyricicoccaceae*	0.23	0.26	0.61 **	0.37
*Oscillospiraceae*	13.11	13.43	15.38	13.48
*Ruminococcaceae*	11.36	10.31	10.86	10.80
*Oscillospirales UCG-010*	0.21	0.18	0.25	0.22
*Coprostanoligenes group*	1.26	1.06	0.67	1.09
*Peptococcaceae*	0.72	0.65	0.52	0.62
*Anaerovoracaceae*	0.26	0.16	0.10 ****	0.16
*Peptostreptococcaceae*	0.58	0.21	0.25	0.53
*Saccharimonadaceae*	0.35	2.06 *	1.07	1.28 *
Uncultured (*Rhodospirillales*)	0.03	0.02	0.17	0.22
*Sutterellaceae*	0.01	0.02	0.03	0.16 ***
*Enterobacteriaceae*	0.69	0.07 *	0.08 *	0.31
*Moraxellaceae*	0.00	0.00	0.00	0.12
*Akkermansiaceae*	0.62	0.67	0.48	0.52

**Table 4:** Average percentage composition of statistically significant (ANOVA test) intestinal microbiota genera and species in CRC-induced animals for the four cohorts studied. Asterisks indicate the level of significant difference compared to the control cohort. Cohort 1: PBS administration; Cohort 2: quercetin administration; Cohort 3: luteolin administration; Cohort 4: xanthohumol administration. Asterisks indicate statistically significant differences (* *p* < 0.05; ** *p* < 0.005; *** *p* < 0.0005; **** *p* < 0.0001).

Genus	Cohort 1 (%)	Cohort 2 (%)	Cohort 3 (%)	Cohort 4 (%)
*Muribaculum*	0.24	0.37 ***	0.56 ****	0.44 ****
*Bilophila*	0.18	0.29	0.80 ***	0.49
*Christensenellaceae R-7*	0.76	0.27	0.08 **	0.66
*Clostridia UCG-014*	4.37	3.64	1.88 **	4.94
*Eubacterium xylanophilum* group	1.40	0.71 *	0.31 **	0.69 *
Uncultured (*Oscillospiraceae*)	3.99	4.84	6.06 *	4.72
Uncultured (*Ruminococcaceae*)	0.45	0.46	1.27 ****	0.98 **
“*Candidatus* Saccharimonas”	0.35	2.06 **	1.07	1.28
*Parasutterella*	0.01	0.02	0.03	0.16 *
*Turicibacter*	0.77	0.46	0.07 **	0.40
*Gastranaerophilales*	0.20	0.25	0.22	0.71 *
Uncultured (*Erysipelotrichaceae*)	0.02	0.13 *	0.06	0.02
**Species**				
*Bacteroides* sp.	0.23	0.27	1.45 **	1.04
*Eubacterium* sp.	0.06	0.33	0.30	0.58 *
*Coprostanoligenes* group				
*UCG-005 metagenome*	0.05	0.13 *	0.08	0.08

## Data Availability

Data (numbers of tumors, hyperplastic Peyer patches, etc.) and materials (tissues maintained in paraformaldehyde) can be obtained from the research group upon request. Publicly available datasets (metagenome sequences) were analyzed in this study, and these data can be found at the NCBI SRA database with access number PRJNA1083865.

## Supplementary Materials

The following supporting information can be downloaded at: https://www.mdpi.com/article/10.3390/nu16081161/s1. Figure S1. Mean number of caecum weight in grams for each cohort in CRC-induced animals (rats 1 to 8 in each cohort). The median value of each cohort is represented as a horizontal line within the corresponding box plots. Figure S2. Mean numbers of colon length in centimeters for each cohort in CRC-induced animals (rats 1 to 8 in each cohort). The median value of each cohort is represented as a horizontal line within the corresponding box plots. Figure S3. Box plots indicating alpha diversity measures of CRC-induced animals using the Chao1 (A), Simpson (B), and Shannon (C) indices. The median value of each cohort is represented as a horizontal line within the corresponding box plots. Figure S4. Heatmap with cluster samples by genus abundance. A dendrogram is used to show how samples are grouped based on genus abundance. Row labels add phylum information. Colors represent standardized abundances (red means high abundance of the given genus, while blue means low abundance).

## References

[1] Imran M., Rauf A., Abu-Izneid T., Nadeem M., Shariati M.A., Khan I.A., Imran A., Orhan I.E., Rizwan M., Atif M. (2019). Luteolin, a Flavonoid, as an Anticancer Agent: A Review. Biomed. Pharmacother..

[2] Devi K.P., Rajavel T., Nabavi S.F., Setzer W.N., Ahmadi A., Mansouri K., Nabavi S.M. (2015). Hesperidin: A Promising Anticancer Agent from Nature. Ind. Crops Prod..

[3] Xi Y., Xu P. (2021). Global Colorectal Cancer Burden in 2020 and Projections to 2040. Transl. Oncol..

[4] Siegel R.L., Miller K.D., Fuchs H.E., Jemal A. (2021). Cancer Statistics, 2021. CA Cancer J. Clin..

[5] Constantinou V., Constantinou C. (2024). Focusing on Colorectal Cancer in Young Adults (Review). Mol. Clin. Oncol..

[6] Kuipers E.J., Grady W.M., Lieberman D., Seufferlein T., Sung J.J., Boelens P.G., van de Velde C.J.H., Watanabe T. (2015). Colorectal Cancer. Nat. Rev. Dis. Primers.

[7] Jemal A., Bray F., Center M.M., Ferlay J., Ward E., Forman D. (2011). Global Cancer Statistics. CA Cancer J. Clin..

[8] Fernández J., García L., Monte J., Villar C.J., Lombó F. (2018). Functional Anthocyanin-Rich Sausages Diminish Colorectal Cancer in an Animal Model and Reduce pro-Inflammatory Bacteria in the Intestinal Microbiota. Genes.

[9] Parmar S., Easwaran H. (2022). Genetic and Epigenetic Dependencies in Colorectal Cancer Development. Gastroenterol. Rep..

[10] Kim U., Lee D.S. (2023). Epigenetic Regulations in Mammalian Cells: Roles and Profiling Techniques. Mol. Cells.

[11] Zeki S.S., Graham T.A., Wright N.A. (2011). Stem Cells and Their Implications for Colorectal Cancer. Nat. Rev. Gastroenterol. Hepatol..

[12] Colussi D., Brandi G., Bazzoli F., Ricciardiello L. (2013). Molecular Pathways Involved in Colorectal Cancer: Implications for Disease Behavior and Prevention. Int. J. Mol. Sci..

[13] Grady W.M., Carethers J.M. (2008). Genomic and Epigenetic Instability in Colorectal Cancer Pathogenesis. Gastroenterology.

[14] Jones S., Chen W.D., Parmigiani G., Diehl F., Beerenwinkel N., Antal T., Traulsen A., Nowak M.A., Siegel C., Velculescu V.E. (2008). Comparative Lesion Sequencing Provides Insights into Tumor Evolution. Proc. Natl. Acad. Sci. USA.

[15] Tsao R. (2010). Chemistry and Biochemistry of Dietary Polyphenols. Nutrients.

[16] Manach C., Scalbert A., Morand C., Rémésy C., Jiménez L. (2004). Polyphenols: Food Sources and Bioavailability. Am. J. Clin. Nutr..

[17] Chaudhuri S., Sengupta B., Taylor J., Pahari B.P., Sengupta P.K. (2013). Interactions of Dietary Flavonoids with Proteins: Insights from Fluorescence Spectroscopy and Other Related Biophysical Studies. Curr. Drug Metab..

[18] González-Vallinas M., González-Castejón M., Rodríguez-Casado A., Ramírez de Molina A. (2013). Dietary Phytochemicals in Cancer Prevention and Therapy: A Complementary Approach with Promising Perspectives. Nutr. Rev..

[19] Li A.-N., Li S., Zhang Y.-J., Xu X.-R., Chen Y.-M., Li H.-B. (2014). Resources and Biological Activities of Natural Polyphenols. Nutrients.

[20] Yahfoufi N., Alsadi N., Jambi M., Matar C. (2018). The Immunomodulatory and Anti-Inflammatory Role of Polyphenols. Nutrients.

[21] Rodríguez-García C., Sánchez-Quesada C., Gaforio J.J. (2019). Dietary Flavonoids as Cancer Chemopreventive Agents: An Updated Review of Human Studies. Antioxidants.

[22] Abotaleb M., Samuel S., Varghese E., Varghese S., Kubatka P., Liskova A., Büsselberg D. (2018). Flavonoids in Cancer and Apoptosis. Cancers.

[23] Chirumbolo S., Bjørklund G., Lysiuk R., Vella A., Lenchyk L., Upyr T. (2018). Targeting Cancer with Phytochemicals via Their Fine Tuning of the Cell Survival Signaling Pathways. Int. J. Mol. Sci..

[24] Kopustinskiene D.M., Jakstas V., Savickas A., Bernatoniene J. (2020). Flavonoids as Anticancer Agents. Nutrients.

[25] Gorlach S., Fichna J., Lewandowska U. (2015). Polyphenols as Mitochondria-Targeted Anticancer Drugs. Cancer Lett..

[26] Perez-Vizcaino F., Fraga C.G. (2018). Research Trends in Flavonoids and Health. Arch. Biochem. Biophys..

[27] Rauf A., Imran M., Khan I.A., ur-Rehman M., Gilani S.A., Mehmood Z., Mubarak M.S. (2018). Anticancer Potential of Quercetin: A Comprehensive Review. Phytother. Res..

[28] Jiang C.H., Sun T.L., Xiang D.X., Wei S.S., Li W.Q. (2018). Anticancer Activity and Mechanism of Xanthohumol: A Prenylated Flavonoid from Hops (*Humulus lupulus* L.). Front. Pharmacol..

[29] Harwood M., Danielewska-Nikiel B., Borzelleca J.F., Flamm G.W., Williams G.M., Lines T.C. (2007). A Critical Review of the Data Related to the Safety of Quercetin and Lack of Evidence of in Vivo Toxicity, Including Lack of Genotoxic/Carcinogenic Properties. Food Chem. Toxicol..

[30] Kawanishi S., Oikawa S., Murata M. (2005). Evaluation for Safety of Antioxidant Chemopreventive Agents. Antioxid. Redox Signal..

[31] Liu H., Zhang L., Li G., Gao Z. (2020). Xanthohumol Protects against Azoxymethane-Induced Colorectal Cancer in Sprague-Dawley Rats. Environ. Toxicol..

[32] Vanhoecke B.W., Delporte F., Van Braeckel E., Heyerick A., Depypere H.T., Nuytinck M., De Keukeleire D., Bracke M.E. (2005). A Safety Study of Oral Tangeretin and Xanthohumol Administration to Laboratory Mice. In Vivo.

[33] Miranda C.L., Stevens J.F., Helmrich A., Henderson M.C., Rodriguez R.J., Yang Y.-H., Deinzer M.L., Barnes D.W., Buhler D.R. (1999). Antiproliferative and Cytotoxic Effects of Prenylated Flavonoids from Hops (*Humulus lupulus*) in Human Cancer Cell Lines. Food Chem. Toxicol..

[34] Lee S.H., Kim H.J., Lee J.S., Lee I.-S., Kang B.Y. (2007). Inhibition of Topoisomerase I Activity and Efflux Drug Transporters’ Expression by Xanthohumol from Hops. Arch. Pharm. Res..

[35] Fernández J., Silván B., Entrialgo-Cadierno R., Villar C.J., Capasso R., Uranga J.A., Lombó F., Abalo R. (2021). Antiproliferative and Palliative Activity of Flavonoids in Colorectal Cancer. Biomed. Pharmacother..

[36] Nejabati H.R., Roshangar L. (2022). Kaempferol: A Potential Agent in the Prevention of Colorectal Cancer. Physiol. Rep..

[37] Al Shoyaib A., Archie S.R., Karamyan V.T. (2020). Intraperitoneal Route of Drug Administration: Should It Be Used in Experimental Animal Studies?. Pharm. Res..

[38] Murota K., Nakamura Y., Uehara M. (2018). Flavonoid Metabolism: The Interaction of Metabolites and Gut Microbiota. Biosci. Biotechnol. Biochem..

[39] Thangaraj K., Natesan K., Settu K., Palani M., Govindarasu M., Subborayan V., Vaiyapuri M. (2018). Orientin Mitigates 1, 2-Dimethylhydrazine Induced Lipid Peroxidation, Antioxidant and Biotransforming Bacterial Enzyme Alterations in Experimental Rats. J. Cancer Res. Ther..

[40] Nowak B., Poźniak B., Popłoński J., Bobak Ł., Matuszewska A., Kwiatkowska J., Dziewiszek W., Huszcza E., Szeląg A. (2020). Pharmacokinetics of Xanthohumol in Rats of Both Sexes after Oral and Intravenous Administration of Pure Xanthohumol and Prenylflavonoid Extract. Adv. Clin. Exp. Med..

[41] Neamtu A., Maghiar T., Alaya A., Olah N., Turcus V., Pelea D., Totolici B.D., Neamtu C., Maghiar A.M., Mathe E. (2022). A Comprehensive View on the Quercetin Impact on Colorectal Cancer. Molecules.

[42] Mishra S., Tripathi A., Chaudhari B.P., Dwivedi P.D., Pandey H.P., Das M. (2014). Deoxynivalenol Induced Mouse Skin Cell Proliferation and Inflammation via MAPK Pathway. Toxicol. Appl. Pharmacol..

[43] Aziz N., Kim M.Y., Cho J.Y. (2018). Anti-Inflammatory Effects of Luteolin: A Review of in Vitro, in Vivo, and in Silico Studies. J. Ethnopharmacol..

[44] Cho Y.C., Kim H.J., Kim Y.J., Lee K.Y., Choi H.J., Lee I.S., Kang B.Y. (2008). Differential Anti-Inflammatory Pathway by Xanthohumol in IFN-γ and LPS-Activated Macrophages. Int. Immunopharmacol..

[45] Chiang M.C., Tsai T.Y., Wang C.J. (2023). The Potential Benefits of Quercetin for Brain Health: A Review of Anti-Inflammatory and Neuroprotective Mechanisms. Int. J. Mol. Sci..

[46] Carbonaro M., Grant G. (2005). Absorption of Quercetin and Rutin in Rat Small Intestine. Ann. Nutr. Metab..

[47] Hughes R.L., Alvarado D.A., Swanson K.S., Holscher H.D. (2022). The Prebiotic Potential of Inulin-Type Fructans: A Systematic Review. Adv. Nutr..

[48] Fernández J., Ledesma E., Monte J., Millán E., Costa P., de la Fuente V.G., García M.T.F., Martínez-Camblor P., Villar C.J., Lombó F. (2019). Traditional Processed Meat Products Re-Designed Towards Inulin-Rich Functional Foods Reduce Polyps in Two Colorectal Cancer Animal Models. Sci. Rep..

[49] Xiao J., Metzler-Zebeli B.U., Zebeli Q. (2015). Gut Function-Enhancing Properties and Metabolic Effects of Dietary Indigestible Sugars in Rodents and Rabbits. Nutrients.

[50] Oteiza P.I., Fraga C.G., Mills D.A., Taft D.H. (2018). Flavonoids and the Gastrointestinal Tract: Local and Systemic Effects. Mol. Asp. Med..

[51] Ashokkumar P., Sudhandiran G. (2011). Luteolin Inhibits Cell Proliferation during Azoxymethane-Induced Experimental Colon Carcinogenesis via Wnt/ β-Catenin Pathway. Investig. New Drugs.

[52] Matsukawa Y., Nishino H., Okuyama Y., Matsui T., Matsumoto T., Matsumura S., Shimizu Y., Sowa Y., Sakai T. (1997). Effects of Quercetin and/or Restraint Stress on Formation of Aberrant Crypt Foci Induced by Azoxymethane in Rat Colons. Oncology.

[53] Deschner E.E., Ruperto J., Wong G., Newmark H.L. (1991). Quercetin and Rutin as Inhibitors of Azoxymethanol-Induced Colonic Neoplasia. Carcinogenesis.

[54] Torrens-Mas M., Alorda-Clara M., Martínez-Vigara M., Roca P., Sastre-Serra J., Oliver J., Pons D.G. (2022). Xanthohumol Reduces Inflammation and Cell Metabolism in HT29 Primary Colon Cancer Cells. Int. J. Food Sci. Nutr..

[55] Sastre-Serra J., Ahmiane Y., Roca P., Oliver J., Pons D.G. (2019). Xanthohumol, a Hop-Derived Prenylflavonoid Present in Beer, Impairs Mitochondrial Functionality of SW620 Colon Cancer Cells. Int. J. Food Sci. Nutr..

[56] Aguirre A.R., Abensur H. (2014). Physiology of Fluid and Solute Transport across the Peritoneal Membrane. J. Bras. Nefrol..

[57] Landete J.M. (2011). Ellagitannins, Ellagic Acid and Their Derived Metabolites: A Review about Source, Metabolism, Functions and Health. Food Res. Int..

[58] Jaganath I.B., Jaganath I.B., Mullen W., Edwards C.A., Crozier A. (2006). The Relative Contribution of the Small and Large Intestine to the Absorption and Metabolism of Rutin in Man. Free Radic. Res..

[59] Marín L., Miguélez E.M., Villar C.J., Lombó F. (2015). Bioavailability of Dietary Polyphenols and Gut Microbiota Metabolism: Antimicrobial Properties. BioMed Res. Int..

[60] Selma M.V., Espín J.C., Tomás-Barberán F.A. (2009). Interaction between Phenolics and Gut Microbiota: Role in Human Health. J. Agric. Food Chem..

[61] Nazzaro F., Fratianni F., De Feo V., Battistelli A., Da Cruz A.G., Coppola R. (2020). Polyphenols, the New Frontiers of Prebiotics.

[62] Makarewicz M., Drożdż I., Tarko T., Duda-Chodak A. (2021). The Interactions between Polyphenols and Microorganisms, Especially Gut Microbiota. Antioxidants.

[63] Zhu Q., Jin Z., Wu W., Gao R., Guo B., Gao Z., Yang Y., Qin H. (2014). Analysis of the Intestinal Lumen Microbiota in an Animal Model of Colorectal Cancer. PLoS ONE.

[64] Yazici C., Wolf P.G., Kim H., Cross T.-W.L., Vermillion K., Carroll T., Augustus G.J., Mutlu E., Tussing-Humphreys L., Braunschweig C. (2017). Race-Dependent Association of Sulfidogenic Bacteria with Colorectal Cancer. Gut.

[65] Le Gall G., Guttula K., Kellingray L., Tett A.J., ten Hoopen R., Kemsley E.K., Savva G.M., Ibrahim A., Narbad A. (2018). Metabolite Quantification of Faecal Extracts from Colorectal Cancer Patients and Healthy Controls. Oncotarget.

[66] Burns M.B., Montassier E., Abrahante J., Priya S., Niccum D.E., Khoruts A., Starr T.K., Knights D., Blekhman R. (2018). Colorectal Cancer Mutational Profiles Correlate with Defined Microbial Communities in the Tumor Microenvironment. PLoS Genet..

[67] Waters J.L., Ley R.E. (2019). The Human Gut Bacteria Christensenellaceae Are Widespread, Heritable, and Associated with Health. BMC Biol..

[68] Qu R., Zhang Y., Ma Y., Zhou X., Sun L., Jiang C., Zhang Z., Fu W. (2023). Role of the Gut Microbiota and Its Metabolites in Tumorigenesis or Development of Colorectal Cancer. Adv. Sci..

[69] Fernández J., Redondo-Blanco S., Gutiérrez-del-Río I., Miguélez E.M., Villar C.J., Lombó F. (2016). Colon Microbiota Fermentation of Dietary Prebiotics towards Short-Chain Fatty Acids and Their Roles as Anti-Inflammatory and Antitumour Agents: A Review. J. Funct. Foods.

[70] Liu N., Zou S., Xie C., Meng Y., Xu X. (2023). Effect of the β-Glucan from Lentinus Edodes on Colitis-Associated Colorectal Cancer and Gut Microbiota. Carbohydr. Polym..

[71] Peck S.C., Denger K., Burrichter A., Irwin S.M., Balskus E.P., Schleheck D. (2019). A Glycyl Radical Enzyme Enables Hydrogen Sulfide Production by the Human Intestinal Bacterium Bilophila Wadsworthia. Proc. Natl. Acad. Sci. USA.

[72] Mancabelli L., Milani C., Lugli G.A., Turroni F., Cocconi D., van Sinderen D., Ventura M. (2017). Identification of Universal Gut Microbial Biomarkers of Common Human Intestinal Diseases by Meta-Analysis. FEMS Microbiol. Ecol..

[73] Wang Y., Nan X., Zhao Y., Jiang L., Wang H., Zhang F., Hua D., Liu J., Yao J., Yang L. (2021). Dietary Supplementation of Inulin Ameliorates Subclinical Mastitis via Regulation of Rumen Microbial Community and Metabolites in Dairy Cows. Microbiol. Spectr..

[74] Lin H., Ma X., Yang X., Chen Q., Wen Z., Yang M., Fu J., Yin T., Lu G., Qi J. (2022). Natural Shikonin and Acetyl-Shikonin Improve Intestinal Microbial and Protein Composition to Alleviate Colitis-Associated Colorectal Cancer. Int. Immunopharmacol..

[75] Weber A.M., Ibrahim H., Baxter B.A., Kumar R., Maurya A.K., Kumar D., Agarwal R., Raina K., Ryan E.P. (2023). Integrated Microbiota and Metabolite Changes Following Rice Bran Intake during Murine Inflammatory Colitis-Associated Colon Cancer and in Colorectal Cancer Survivors. Cancers.

[76] Zhang J., Chen Z., Lu Y., Tu D., Zou F., Lin S., Yu W., Miao M., Shi H. (2021). A Functional Food Inhibits Azoxymethane/Dextran Sulfate Sodium-Induced Inflammatory Colorectal Cancer in Mice. Onco. Targets Ther..

[77] Bosshard P.P., Zbinden R., Altwegg M. (2002). Turicibacter Sanguinis Gen. Nov., Sp. Nov., a Novel Anaerobic, Gram-Positive Bacterium. Int. J. Syst. Evol. Microbiol..

[78] Chung Y., Ryu Y., An B.C., Yoon Y.S., Choi O., Kim T.Y., Yoon J., Ahn J.Y., Park H.J., Kwon S.K. (2021). A Synthetic Probiotic Engineered for Colorectal Cancer Therapy Modulates Gut Microbiota. Microbiome.

[79] Zackular J.P., Baxter N.T., Iverson K.D., Sadler W.D., Petrosino J.F., Chen G.Y., Schloss P.D. (2013). The Gut Microbiome Modulates Colon Tumorigenesis. mBio.

[80] Jahani-Sherafat S., Alebouyeh M., Moghim S., Amoli H.A., Ghasemian-Safaei H. (2018). Role of Gut Microbiota in the Pathogenesis of Colorectal Cancer: A Review Article. Gastroenterol. Hepatol. Bed Bench.

[81] Cipe G., Idiz U.O., Firat D., Bektasoglu H. (2015). Relationship Between Intestinal Microbiota and Colorectal Cancer. World J. Gastrointest. Oncol..

