# Supplementary material for: Antitumor Effect and Gut Microbiota Modulation by Quercetin, Luteolin, and Xanthohumol in a Rat Model for Colorectal Cancer Prevention
Source: Nutrients. 2024 Apr 13;16(8):1161. doi: 10.3390/nu16081161 (PMC11054239; doi:10.3390/nu16081161)
Supplement: Supplementary file 1 [file nutrients-16-01161-s001.zip › nutrients-2938316-supplementary.pdf]

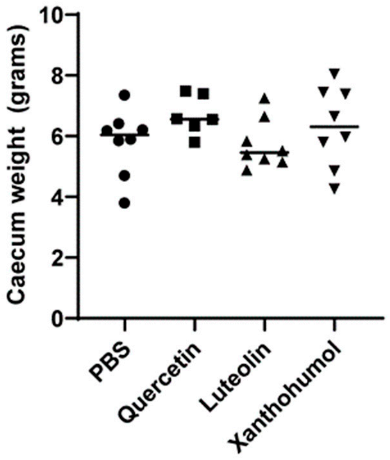

Supplementary Figure S1. Mean number of caecum weight in grams for each cohort in CRC-induced animals (rats 1 to 8 in each cohort). The median value of each cohort is represented as a horizontal line within the corresponding box plots.

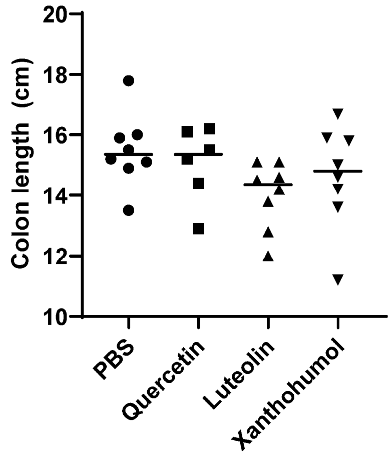

Supplementary Figure S2. Mean numbers of colon length in centimeters for each cohort in CRC-induced animals (rats 1 to 8 in each cohort). The median value of each cohort is represented as a horizontal line within the corresponding box plots.

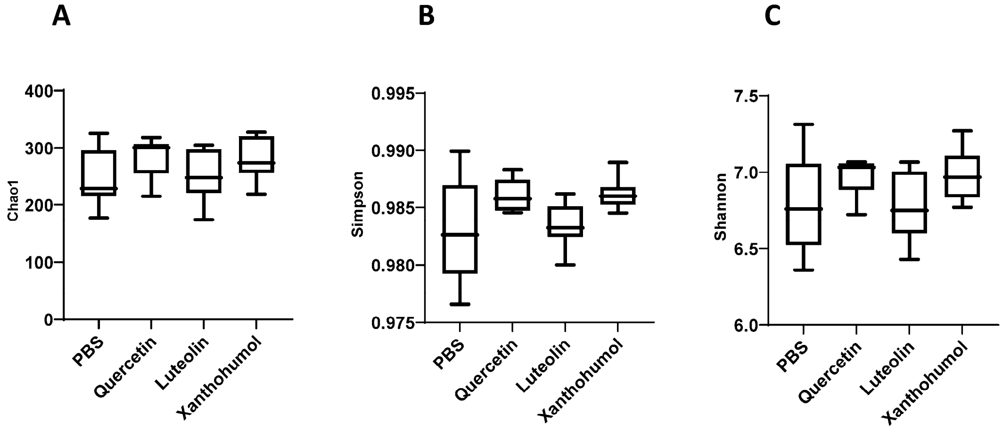

Supplementary Figure S3. Boxplots indicating Alpha diversity measures of CRC-induced animals using the Chao1 (A), Simpson (B) and Shannon (C) indices. The median value of each cohort is represented as a horizontal line within the corresponding box plots.

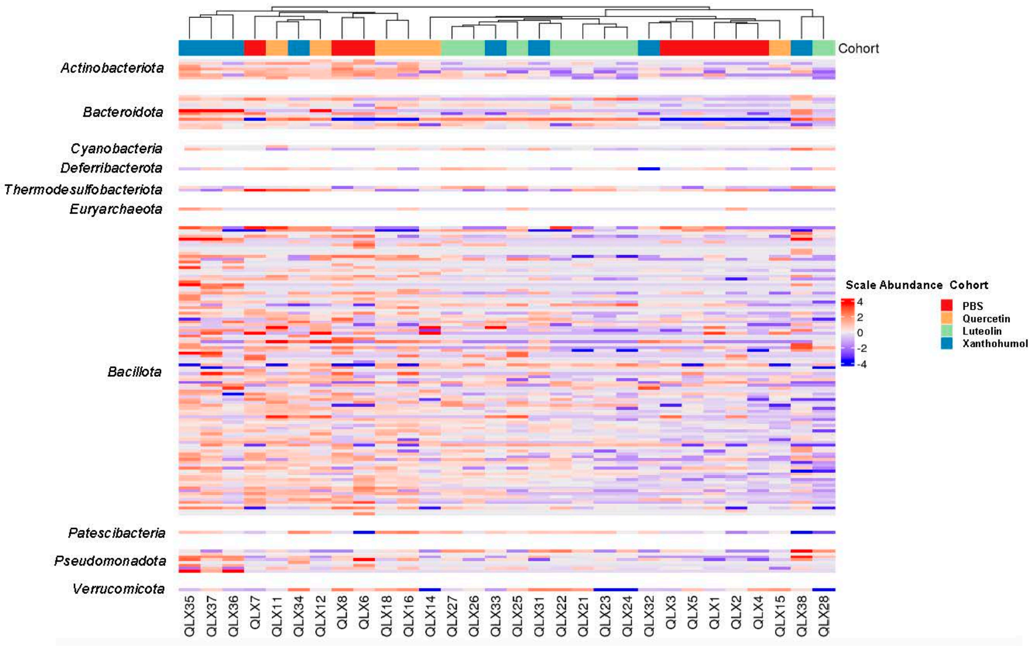

Supplementary Figure S4. Heatmap with cluster samples by genus abundance. A dendogram is used to show how samples are grouped based on genus abundance. Row labels add phylum information. Colors represent standardized abundances (red means high abundance of the given genus, while blue means low abundance).
